# Work withdrawal behavior and its associations with perceived stress and work-life balance among nurses: a multicenter cross-sectional study

**DOI:** 10.3389/fpubh.2025.1708574

**Published:** 2025-12-17

**Authors:** Wenjuan Zhou, Heyu Chen, Dabing Dai, Junrong Ye, Yuanyuan Song, Hongmei Luo, Yu Xu

**Affiliations:** 1 Department of Ophthalmology, West China Hospital of Sichuan University, Chengdu, China; 2 Department of Critical Care Medicine, West China Hospital of Sichuan University, Chengdu, China; 3 Department of Nursing Administration, Affiliated Brain Hospital of Guangzhou Medical University, Guangzhou, China

**Keywords:** influencing factors, nurses, perceived stress, work withdrawal, work-life balance

## Abstract

**Background:**

Work withdrawal behavior adversely affects both individual nurses' career development and team performance. However, the current status of work withdrawal behavior among nurses in China and its relationship with perceived stress and work-life balance has received limited attention.

**Objective:**

This study aims to examine the current status of work withdrawal behavior among nurses in Chinese hospitals and to analyze its associations with perceived stress and work-life balance.

**Methods:**

A cross-sectional survey was conducted from February to March 2025 using convenience sampling to recruit 2,707 nurses from 203 primary, secondary, and tertiary hospitals across 27 provinces in China. Validated scales were used to assess nurses' work withdrawal behavior, perceived stress, and work-life balance. Univariate analysis and multiple linear regression analysis were conducted to examine factors associated with work withdrawal behavior.

**Results:**

Among 2,594 valid questionnaires (response rate: 95.8%), the mean Work Withdrawal Behavior Scale score was 19.14 (*SD* = 6.90). Multiple linear regression revealed that male gender, older age, working in intensive care units or emergency departments, lower monthly income, longer overtime hours, higher perceived stress, and poorer work-life balance were significantly associated with increased work withdrawal behaviors (all *p* < 0.05). Among these, perceived stress (β = 0.291) and work-life balance (β = −0.201) were the strongest predictors.

**Conclusion:**

Work withdrawal behaviors are observable among Chinese nurses, with psychological withdrawal aspects showing relatively higher manifestation. Among the range of associated factors, increased perceived stress and poorer work-life balance were the most salient. Hospitals should therefore implement adequate organizational support, regular psychological screenings, and counseling services to alleviate stress and improve work-life balance, thereby reducing work withdrawal behaviors.

## 1 Introduction

Work withdrawal behavior refers to passive behavioral responses through which individuals evade work tasks when facing stress or job dissatisfaction (1, 2). Its manifestations include daydreaming, work procrastination, absenteeism, tardiness, early departure, and misuse of organizational resources (1, 2). For instance, frequent absences or leaving work early can place a greater burden on remaining team members by increasing their workload, potentially reducing team efficiency and, in healthcare settings, undermining the quality of care and patient outcomes. Additionally, daydreaming and procrastination have been shown to disrupt workflow and stifle creativity and productivity (3, 4). Therefore, recognizing and addressing work withdrawal behaviors is crucial in healthcare settings for maintaining team effectiveness, promoting employee wellbeing, and safeguarding high-quality patient care.

Work withdrawal behaviors often reflect negative psychological states and may signal underlying psychological issues. Research shows that frequent work withdrawal is associated with decreased self-efficacy, which can undermine an individual's confidence in coping with challenges and hinder their career development (5). As essential healthcare providers, nurses are frequently exposed to high-intensity workloads and disrupted circadian rhythms, increasing their likelihood of exhibiting work withdrawal. A cross-sectional study of 533 nurses in Hunan province, China, revealed that over 40% of nurses exhibited work withdrawal behaviors (2). However, there remains a significant dearth of research investigating these behaviors and their associated factors, especially those amenable to intervention, among Chinese nurses.

Perceived stress, the subjective appraisal of one's work environment as demanding or overwhelming (6), is a central concept in understanding work withdrawal among nurses. According to the Conservation of Resources Theory, individuals tend to protect their valued psychological and physical resources. Nurses experiencing high levels of perceived stress continuously deplete their psychological resources due to work demands, resulting in a significant resource imbalance (7, 8). Such an imbalance may lead to increased work withdrawal behavior as nurses seek to restore their psychological resource balance. This pathway is supported by empirical evidence linking higher perceived stress to a diminished sense of professional identity (9) and increased emotional exhaustion (10), both of which positively correlated with work withdrawal behavior (11). Corroborating this, a study in the German healthcare context reported that heightened stress led to increased work withdrawal behavior and occupational burnout among medical staff (12).

Work-life balance, conceptualized as the equitable allocation of an individual's resources, energy, and time between professional and personal domains, plays a critical role in nurse wellbeing and retention (13, 14). Similarly, the Conservation of Resources Theory suggests that when nurses perceive an unequal distribution of resources between work and life, they may feel threatened and resort to withdrawal behaviors to protect their psychological resources. This is consistent with research showing that an imbalance between work and life can result in work-family conflict (15), which is positively associated with work withdrawal behavior (16). Consequently and as empirically demonstrated among healthcare professionals, a significant negative correlation exists between work-life balance and work withdrawal behavior (17).

Although previous research has predominantly examined the impact of socio-demographic factors on work withdrawal behavior among Chinese nurses (2, 16, 18, 19), the independent roles of perceived stress and work-life balance remain underexplored. This gap limits systematic explanation and highlights inadequate cognitive frameworks. Therefore, this study employed a multi-center, cross-sectional design to examine the current status of work withdrawal behavior among nurses working in Chinese hospitals and to analyze its associations with perceived stress and work-life balance. By elucidating these multi-level determinants, this study seeks to advance the recognition of occupational burnout as a systemic and inform integrated, multi-level support systems to facilitate sustainable work-life balance and mitigate burnout.

Based on the above, we formulate the following hypothesis:

Hypothesis 1: A significant positive correlation exists between perceived stress and work withdrawal behaviors among nurses in Chinese hospitals.

Hypothesis 2: A significant negative correlation exists between work-life balance and work withdrawal behavior among nurses in Chinese hospitals.

## 2 Methods

### 2.1 Study design and participants

This study adopted a multi-center cross-sectional survey design. Data were collected between February and March 2025 from 203 primary, secondary, and tertiary hospitals across 27 provinces in China using convenience sampling method, to ensure a representative sample of nurses from the three-tiered healthcare system. The study adhered strictly to the Strengthening the Reporting of Observational Studies in Epidemiology (STROBE) statement to ensure transparency and completeness (20). Inclusion criteria were: (1) holding a valid registered nurse license; (2) having at least 1 year of clinical experience and currently practicing in the relevant department; (3) serving as a clinical nurse; (4) providing voluntary participation and informed consent, and (5) working in primary, secondary or tertiary hospitals. Exclusion criteria included: (1) temporary absence due to illness, personal leave, or external training; (2) current enrollment in educational programs, internships, or formal training; and (3) primary responsibilities in teaching or research with limited engagement in clinical practice.

### 2.2 Sample size

The sample size was estimated using the formula for cross-sectional surveys recommended by Wang et al. (21): *n* = (*Z*^2^ × σ^2^)/e^2^. The population standard deviation was estimated as σ = 4.26 based on a previous study (22). With a 95% confidence level (*Z* = 1.96) and a tolerable margin of error (*e* = 0.2), the calculation yields a minimum required sample size of 1,743 participants. To allow for a 20% rate of nonresponse or invalid questionnaires, 2,179 participants were needed.

### 2.3 Data collection

Between February and March 2025, a questionnaire comprising established and validated scales was administered to collect data. The questionnaire was built and distributed via the Questionnaire Star online platform. Prior to disseminating the survey, the principal investigator (DDB) contacted the directors of nursing departments of each potential participating hospital to ascertain their willingness to participate. Upon obtaining institutional approval, the survey link was shared with head nurses of relevant clinical departments, who subsequently forwarded it to nurses. The first page of the online questionnaire provided detailed information about the study objectives, inclusion and exclusion criteria, and an informed consent statement. Potential participants who met the eligibility criteria and voluntarily agreed to participate provided electronic consent by selecting the “agree” option, which then directed them to the questionnaire. Those who did not meet the eligibility criteria or declined participation could exit the survey platform immediately. The estimated time for completing the survey ranged from 8 to 15 min. Participants were assured of the voluntary and anonymous nature of the survey and encouraged to respond based on their genuine experiences and perceptions. To ensure data quality, all questionnaire items were set as mandatory, and only one submission per IP address was permitted. During data cleaning process, the researcher eliminated questionnaires exhibiting obvious patterns of invalidity (e.g., selecting the same response for all items), illogical answers (e.g., an age of 27 with 30 years of work experience), or completion times of < 180 s.

### 2.4 Measurement tools

#### 2.4.1 General characteristics questionnaire

Based on a literature review and group discussions, the researchers designed a general characteristics questionnaire tailored to the study objectives. The questionnaire comprised 16 items, including gender, age, education background, marital status, position, title, hospital level, department, years of work experience, number of children, care responsibilities for infants and young children or cohabitation status, frequency of night shifts, monthly income, average overtime working hours per week, sick leave taken in the past year, and presence of chronic diseases. In accordance with the characteristics of nursing roles in China, positions were classified into two groups: frontline clinical nurses (those who deliver direct patient care at the bedside) and non-frontline clinical nurses (those responsible for patient admissions and discharges, management of unit supplies, and administrative duties within the unit).

#### 2.4.2 The work withdrawal behavior scale

The work withdrawal behavior scale, originally developed by Lehman et al. (23) in 1992, is designed to assess employees' work withdrawal behaviors over the preceding year. The scale was subsequently translated into Chinese and culturally adapted by Tian et al. (24) in 2018, demonstrating excellent reliability, as evidenced by a Cronbach's α coefficient of 0.876. The Chinese version comprises two dimensions: psychological withdrawal behavior and physical withdrawal behavior, with a total of 12 items. Responses are recorded on a five-point Likert scale ranging from “Never” (1 point) to “Always” (5 points). The total score ranges from 12 to 60, with higher scores indicating a higher level of work withdrawal behavior. In the current study, the scale demonstrated excellent internal consistency, with a Cronbach's α coefficient of 0.903.

#### 2.4.3 Perceived stress scale

The perceived stress scale, originally developed by Cohen et al. (25) in 1983, was designed to assess an individual's subjective perception of stress levels. The scale was subsequently translated and validated in Chinese by Wang et al. (26) in 2015, demonstrating excellent reliability with a Cronbach's α coefficient of 0.91. The Chinese version of the scale consists of 10 items rated on a five-point Likert scale ranging from “Never” (0 points) to “Very often” (4 points). The total score ranges from 0 to 40, with higher scores indicating greater perceived stress. In the current study, the scale exhibited good internal consistency, with a Cronbach's α coefficient of 0.853.

#### 2.4.4 Work-life balance scale

The work-life balance scale was developed by Huang (27) in 2017 to measure the work-life balance status of employees. The Cronbach's α coefficient reported in the original study was 0.86 (27). The scale consists of 17 items rated on a six-point Likert scale ranging from “Not at all” (1 point) to “Completely” (6 points). The total score ranges from 17 to 102, with higher scores indicating a better work-life balance. The Cronbach's α coefficient for the scale in this study was 0.954.

### 2.5 Ethics approval

This study was approved by the Institutional Biomedical Ethics Committee of the West China Hospital, Sichuan University (Approval No. 20242520). All participants were fully informed and provided electronic consent prior to the survey. All data were anonymized before analysis to protect participant privacy.

### 2.6 Statistical analysis

Statistical analyses were performed using SPSS version 26.0. Categorical data are presented as frequencies and percentages. Continuous variables such as age and years of work were categorized according to conventional clinical or research groupings to illustrate their distribution patterns. Other continuous variables that followed a normal distribution are reported as means ± standard deviations. Group comparisons were conducted using independent samples *t*-tests for two groups and one-way ANOVA for multiple groups, with statistically significant set at *p* < 0.05. Pearson correlation analysis was employed to examine the relationships between perceived stress, work-life balance, and nurses' work withdrawal behavior. Multivariate analysis was performed using multiple linear stepwise regression, with a significance level set at α = 0.05.

## 3 Results

### 3.1 General characteristics of the participants

A total of 2,707 questionnaires were collected. After verification by two researchers, 113 were excluded, resulting in 2,594 valid questionnaires and a response rate of 95.8%. The majority of participants were frontline clinical nurses (87.5%), female (86.2%), and employed in tertiary hospitals (86.2%), with a mean age of ~32 years. A bachelor's degree was the most prevalent educational attainment (76.5%), and 57.9% held junior-level professional titles. Significant differences (*p* < 0.05) in work withdrawal behavior scores were observed across the following variables, including gender, age, marital status, position, title, department, years of work experience, number of children, caregiving or cohabitation with infants, monthly income, average overtime working hours per week, sick leave taken in the past year, and presence of a chronic disease (Table 1).

**Table 1:** General characteristics of the participants (*n* = 2,594).

**Characteristics**		***n* (%)/*M* ±SD**	**Work withdrawal behavior (*M* ±SD)**	***Statistics***	***p*-Value**
Gender	Male	359 (13.8)	20.43 ± 7.92	*t* = 3.390	0.001^*^
	Female	2,235 (86.2)	18.93 ± 6.70		
Age (years)	—	32.33 ± 6.37	—	*r* = 0.076	< 0.001^*^
Education background	Associate degree or lower	489 (18.9)	19.28 ± 8.16	*F =* 1.581	0.206
	Bachelor's degree	1,984 (76.5)	19.04 ± 6.60		
	Master's degree or higher	121 (4.6)	20.14 ± 6.04		
Marital status	Unmarried	869 (33.5)	18.44 ± 6.79	*F =* 7.128	0.001^*^
	Married	1,663 (64.1)	19.46 ± 6.90		
	Divorced or widowed	62 (2.4)	20.27 ± 7.60		
Position	Non-frontline clinical nurse	324 (12.5)	19.77 ± 5.79	*t* = 2.039	0.042^*^
	Frontline clinical nurse	2,270 (87.5)	19.05 ± 7.04		
Title	Junior	1,503 (57.9)	18.57 ± 7.19	*F =* 13.434	< 0.001^*^
	Intermediate	958 (36.9)	19.79 ± 6.41		
	Senior	133 (5.2)	20.81 ± 6.28		
Hospital level	Tertiary	2,236 (86.2)	19.08 ± 6.93	*t* = −1.139	0.255
	Secondary and primary	358 (13.8)	19.52 ± 6.69		
Department	General medical departments	1,272 (49.0)	18.54 ± 6.42	*t* = −4.353	< 0.001^*^
	Intensive care unit and emergency department	1,322 (51.0)	19.71 ± 7.29		
Years of work experience	—	10.08 ± 6.86	—	*r =* 0.056	0.005^*^
Number of children	0	1,112 (42.9)	18.62 ± 6.83	*F =* 6.165	0.002^*^
	1	986 (38.0)	19.39 ± 6.70		
	≥2	496 (19.1)	19.80 ± 7.36		
Caregiving or cohabitation with infants	No	1,360 (52.4)	18.83 ± 6.88	*t* = −2.362	0.018^*^
	Yes	1,234 (47.6)	19.47 ± 6.90		
Frequency of night shift (times/month)	0–3	742 (28.6)	19.09 ± 5.94	*F =* 0.686	0.561
	4–6	592 (22.8)	18.93 ± 6.99		
	7–9	728 (28.1)	19.09 ± 7.05		
	≥10	532 (20.5)	19.50 ± 7.77		
Monthly income (yuan)	≤ 5,000	538 (20.7)	19.40 ± 7.96	*F =* 3.797	0.010^*^
	5,001–8,000	1,020 (39.4)	19.31 ± 7.25		
	8,001–11,000	584 (22.5)	19.35 ± 6.29		
	>11,000	452 (17.4)	18.15 ± 5.18		
Average overtime working hours (h/week)	0–3	1,300 (50.1)	18.15 ± 6.24	*F =* 19.454	< 0.001^*^
	4–6	597 (23.0)	19.82 ± 6.79		
	7–9	357 (13.8)	20.07 ± 8.03		
	≥10	340 (13.1)	20.74 ± 7.61		
Sick leave taken in the last year	No	2,263 (87.2)	19.02 ± 6.84	*t* = −2.200	0.028^*^
	Yes	331 (12.8)	19.92 ± 7.26		
Presence of chronic disease	No	2,150 (82.9)	18.87 ± 6.85	*t* = −4.301	< 0.001^*^
	Yes	444 (17.1)	20.41 ± 6.98		

^*^p < 0.05.

### 3.2 Scores on work-life balance, perceived stress, and work withdrawal behavior of the participants

In this study, the total mean scores of nurses' work-life balance, perceived stress, and work withdrawal behavior were 73.29 ± 16.74, 29.16 ± 6.36, and 19.14 ± 6.90, respectively. Specific scores of each scale are shown in Table 2.

**Table 2:** Descriptive analyses of participants' work-life balance, perceived stress, and work withdrawal behavior (*n* = 2,594).

**Variable**	**Items**	**Score (*M* ±*SD*)**	**Score of items (*M* ±*SD*)**
Work-life balance	17	73.29 ± 16.74	4.31 ± 0.98
Perceived stress	10	29.16 ± 6.36	2.91 ± 0.64
Work withdrawal behavior	12	19.14 ± 6.90	1.60 ± 0.58
Psychological withdrawal	8	14.12 ± 5.20	1.76 ± 0.65
Physical withdrawal	4	5.02 ± 2.22	1.26 ± 0.56

### 3.3 Correlation analysis of work-life balance, perceived stress, and work withdrawal behavior among the participants

As shown in Table 3, work withdrawal behaviors among the participants were positively correlated with perceived stress (*r* = 0.337, *p* < 0.01) and negatively correlated with work-life balance (*r* = −0.263, *p* < 0.01).

**Table 3:** Correlation analysis of work-life balance, perceived stress, and work withdrawal behavior among the participants (*n* = 2,594, *r* value).

**Variable**	**Work-life balance**	**Perceived stress**	**Work withdrawal behavior**
Work-life balance	1.000	—	—
Perceived stress	−0.187^*^	1.000	—
Work withdrawal behavior	−0.263^*^	0.337^*^	1.000

^*^p < 0.01.

### 3.4 Multiple linear regression analysis of work withdrawal behaviors among the participants

This study utilized a linear regression model to analyze the data, with the total score of work withdrawal behavior as the dependent variable. Independent variables were selected based on their statistical significance in univariate analyses. The linearity assumption was verified using scatter plots, and multicollinearity was assessed via the variance inflation factor (VIF), which ranged from 1.0 to 1.2, indicating no substantial multicollinearity and supporting the robustness of the model specification. The regression results demonstrated that several factors were significantly associated with more pronounced work withdrawal behavior: male gender, older age (β = 0.092, *p* < 0.001), working in the intensive care unit or emergency department (β = 0.037, *p* = 0.039), lower monthly income, longer average weekly overtime hours (β = 0.038, *p* = 0.036), and higher perceived stress (β = 0.291, *p* < 0.001). Conversely, a better work-life balance was associated with less evident work withdrawal behavior (Table 4).

**Table 4:** Results of multiple linear regression analysis of work withdrawal behaviors among the participants (*n* = 2,594).

**Variable**	***B***	**SE**	**β**	***t***	***p*-Value**	**95% confidence interval**
Content	14.021	1.110		12.626	< 0.001	11.843–16.198
**Gender**						
Male	Reference					
Female	−1.721	0.363	−0.086	−4.738	< 0.001	−2.434 to −1.009
Age	0.099	0.021	0.092	4.825	< 0.001	0.059–0.140
**Department**						
General medical departments	Reference					
Intensive care unit and emergency department	0.516	0.251	0.037	2.060	0.039	0.025–1.008
**Monthly income**						
≤ 5,000	Reference					
>11,000	−0.703	0.349	−0.039	−2.015	0.044	−1.388 to −0.019
**Average overtime working hours**						
0–3	Reference					
7–9	0.754	0.360	0.038	2.094	0.036	0.048–1.460
Perceived stress	0.316	0.020	0.291	15.907	< 0.001	0.277–0.355
Work-life balance	−0.083	0.008	−0.201	−10.864	< 0.001	−0.098 to −0.068

B = unstandardized coefficient; β = standardized coefficient; SE = standard error, R = 0.415, R^2^ = 0.172, adjusted R^2^ = 0.170; the VIFs ranged from 1.0 to 1.2, below the threshold of 5; F = 76.928, *p* < 0.01.

## 4 Discussion

This study investigated the current status of work withdrawal behavior among nurses across 27 provinces in China and analyzed its relationships with perceived stress and work-life balance. The mean score per item for withdrawal behavior was 1.60 ± 0.58, notably lower than the score of 1.97 ± 0.53 reported in a previous study conducted during the COVID-19 pandemic (16). This difference may be attributed to the substantially increased workload and heightened pressure associated with infection prevention during that period. Furthermore, our findings indicated that psychological withdrawal is more prevalent than physical withdrawal among Chinese nurses. Psychological withdrawal is characterized primarily by thoughts of absence, daydreaming, and engaging in non-work-related conversations with coworkers (22). Previous studies have indicated that high-intensity nursing work coupled with a lack of organizational support can lead to feelings of helplessness and burnout. These factors increase the consumption of psychological resources, subsequently contributing to psychological withdrawal among nurses (28–30). Furthermore, the average nurse-patient ratio in Chinese hospitals is ~1:8 during the day and can reach 1:23 at night (31), creating an occupational environment of overload. In Chinese culture, the value placed on “sticking to one's job” may result in nurses fearing negative feedback or financial repercussions for exhibiting physical withdrawal. Consequently, nurses may opt for psychological withdrawal as a coping strategy.

This study revealed that male nurses exhibited higher levels of work withdrawal behavior than their female counterparts, consistent with the findings of Wang et al. (18). Nursing has traditionally been viewed as a “female-dominated” profession, and male nurses often encounter stereotypical gender roles (32), which may undermine professional identity and increase susceptibility to withdrawal under external pressures (33). Furthermore, male nurses may face ambiguous career development paths within clinical nursing (34), contributing to lower perceived professional benefits (35) and thus exacerbating withdrawal tendencies. To mitigate these issues, nursing managers should implement gender-inclusive training and improve career planning and development opportunities for male nurses.

The findings of this study indicate that older nurses are more likely to exhibit work withdrawal behavior, a result consistent with previous research (2). This pattern can be interpreted through the theoretical lenses of continuance commitment and organizational embeddedness. Older nurses, who typically possess higher sunk costs (e.g., pensions, seniority) and greater non-work responsibilities (such as mortgages or children's education), face considerable personal and economic barriers to resignation (36). As a result, they tend to adopt a passive withdrawal strategy—characterized by psychological disengagement, reduced extra-role contributions, or minimal task compliance—as a means of managing burnout without sacrificing job security. In contrast, other studies suggest that younger nurses are more inclined to pursue actual turnover when dissatisfied (37, 38), reflecting their higher career mobility and lower organizational embeddedness. This divergence underscores that turnover and withdrawal represent distinct coping strategies adopted by different age groups in response to job dissatisfaction. These findings carry important implications for nursing management. A uniform retention strategy is unlikely to be effective. Instead, interventions designed to reduce turnover among younger nurses should emphasize enhanced onboarding processes, mentorship programs, and career advancement opportunities. For older, experienced nurses, strategies to reduce withdrawal behavior should focus on mitigating burnout, fostering respect and autonomy, and implementing job redesign efforts to re-engage them and leverage their extensive clinical experience.

This study found that nurses working in intensive care units and emergency departments reported higher work withdrawal behavior than those in general medical departments, consistent with the results of related studies (18). Nurses in high-stress settings care for critically ill patients with complex, fluctuating conditions and experience high mortality rates. These challenges demand exceptional professional skills, acute clinical judgment, and decisive action, which can result in significant learning pressure and heavy workloads. Prolonged exposure to such stressful environments often leads to emotional exhaustion and burnout (39, 40). Moreover, the unpredictable nature of nursing tasks in intensive care units and emergency departments, including the need for emergent resuscitations and frequent night shifts, disrupts normal physiological rhythms. This disruption can leave nurses in a state of chronic fatigue for extended periods. Additionally, the incidence of medical disputes in these two departments reached as high as 73% (41) and 77% (42), respectively. Nurses in these environments endure significant psychological stress, as they frequently interact with emotionally distressed family members. The disparity between the high levels of stress encountered and nurses' low tolerance for such stress can lead them to engage in withdrawal behaviors, such as absenteeism, passivity, and even resignation. Therefore, nursing managers should enhance humanistic care and provide more substantial organizational support to staff in these high-pressure departments to alleviate work withdrawal behavior.

Furthermore, the study revealed that nurses with higher monthly income reported lower levels of work withdrawal behavior compared to their lower-income counterparts, aligning with the findings of Xiong et al. (2). A study has shown that higher salaries correlate with a decreased intention to leave the nursing profession (43), suggesting that a perceived mismatch between effort and reward may foster organizational dissatisfaction among nurses and contribute to work withdrawal. Additionally, low income can induce financial insecurity, compelling nurses to devote extra energy to part-time jobs or maintaining family finances, thus triggering work withdrawal behavior. To address this, managers should consider implementing a tiered compensation system aligned with work intensity and seniority, ensuring equitable reward for work input, and offering financial planning support to low-income nurses to stabilize their financial situation and reduce work withdrawal behavior.

With respect to overtime working hours, nurses averaged 7–9 h of overtime per week showed higher work withdrawal behavior than those who worked 0–3 h. This may stem from the fact that nurses who frequently work overtime are at a higher risk of emotional exhaustion and burnout (44, 45). In contrast, nurses with fewer overtime hours typically experience lighter workloads and less resource depletion, resulting in lower levels of work withdrawal behavior. In this study, those logging an average of 7–9 h of overtime were predominantly frontline clinical nurses with longer work tenures, resulting in heavier workloads and greater resource consumption. Additionally, extended hours of overtime can trigger severe fatigue, leading to continuous resource depletion without adequate recovery, thereby inducing work withdrawal behavior. Thus, it is advisable for managers to develop a tiered compensation system that accommodates nurses' varying overtime working hours, offering appropriate incentives for hours beyond the standard.

The results of this study revealed a positive correlation between perceived stress and work withdrawal behavior among Chinese nurses, thereby supporting our hypothesis and aligning with prior research (19). The high-stress environment in which nurses work can accelerate emotional exhaustion, leading to psychological resource depletion and burnout (46, 47). The Conservation of Resources Theory suggests that individuals who experience a continuous loss of psychological resources without adequate replenishment instinctively adopt withdrawal behaviors to minimize further loss (7, 8). Prolonged exposure to high-intensity stress can also lead to somatic symptoms such as headaches and gastrointestinal issues (48), prompting nurses to engage in withdrawal behaviors such as absenteeism or requesting reassignment to self-protection. Therefore, promoting positive coping strategies for stress management among nurses is essential. As previous studies demonstrate that different stressors exert varying effects on work withdrawal behavior (49, 50), highlighting the need for nursing managers to comprehensively identify these stressors and implement targeted measures to alleviate them.

Multiple linear regression analysis further demonstrated a significant negative association between work-life balance and work withdrawal behavior among Chinese nurses, thus supporting our hypothesis. Lower work-life balance scores correlated with higher levels of work withdrawal behavior, consistent with earlier findings (16). Nurses operate within a high-intensity, high-stress environment where competing work and family demands can lead to resource depletion, resulting in work withdrawal behavior as a way to conserve their remaining personal resources (16). Effective work-life balance enables nurses to allocate their time and energy more efficiently, reducing role conflict. Improved work-life balance is associated with less psychological distress, stabilized emotional states, and higher job satisfaction, ultimately diminishing stress-induced withdrawal behaviors (19, 51). Studies have shown that a supportive work environment can promote work-life balance and increase nurses' retention (52, 53), thus reducing work withdrawal behavior. To stabilize the nursing workforce and improve care quality, managers should assist nurses in achieving a better work-life balance by optimizing scheduling, providing family support, and strengthening psychological health interventions.

This study has several limitations. First, the data for this study were derived from participants' self-reports, which may lead to recall bias and social desirability bias. For instance, participants might remember low-frequency high-pressure events (e.g., conflicts with family members) more vividly, potentially leading to overestimation of work withdrawal behavior. Conversely, in the hierarchical work environment of Chinese hospitals, nurses may tend to provide answers that conform to social expectations or that they find more acceptable, leading to an underestimation of work withdrawal behavior. Although measures have been taken to ensure anonymity and voluntary participation, eliminating self-report bias remains a challenge. Future studies may consider including objective indicators or the dynamic assessments of work withdrawal behavior to improve data accuracy. Second, the convenience sampling method was used to select the sample in this study, which may have resulted in a sample that was not representative of the broader population. Certain groups in this study (e.g., Southwest and Northwest regions) had a higher percentage of nurses. Future research should adopt stratified sampling to ensure proportional regional representation and enable cross-regional comparisons of work withdrawal behavior. Finally, the cross-sectional design precludes causal inferences between perceived stress, work-life balance, and work withdrawal behavior. Future longitudinal or cohort studies are needed to establish temporal precedence and explore additional relevant variables (e.g., team climate or personality traits) to clarify the specific mechanisms underlying these relationships.

## 5 Conclusion

In this study, 2,594 nurses were surveyed and work withdrawal behaviors were observed, with psychological withdrawal demonstrating greater prominence. Factors such as male gender, older age, employment in intensive care units or emergency departments, lower monthly income, longer overtime working hours, higher scores of perceived stress and lower scores of work-life balance were found significantly associated with increased work withdrawal behaviors a. These findings provide a solid foundation for healthcare organizations to develop targeted interventions to reduce work withdrawal behaviors among nursing staff.

## 6 Implications for nursing management

To further reduce work withdrawal behaviors among nurses, management should optimize its strategies. First, managers should pay special attention to high-risk groups, including older and male, those with lower income, extended overtime hours, and those working in intensive care unit and emergency departments. Regular assessments of perceived stress, work-life balance, and other mental health screenings should be conducted for early identification of those at high risk for work withdrawal. Second, it is essential to optimize the work environment to foster a positive atmosphere. For instance, establishing emotional stress reduction rooms and providing access to counselors can offer one-on-one guidance tailored to the specific triggers influencing nurses' psychological withdrawal. Furthermore, managers should dynamically track indicators of withdrawal behavior—such as absenteeism, turnover intention, and job satisfaction—and intervene promptly when there are upward trends in withdrawal indicators within the department. Additionally, offering more promotional opportunities and specialty training for nurses with low withdrawal behaviors can help maintain their work motivation. Collectively, these strategies are essential to maintaining a long-term stable nursing team performance, which will ensure that patients receive consistent, high-quality care.

## Data Availability

The raw data supporting the conclusions of this article will be made available by the authors, without undue reservation.
